# Maize-Pathogen Interactions: An Ongoing Combat from a Proteomics Perspective

**DOI:** 10.3390/ijms161226106

**Published:** 2015-11-30

**Authors:** Olga Pechanova, Tibor Pechan

**Affiliations:** 1 Mississippi State Chemical Laboratory, Mississippi State University, Mississippi State, MS 39762, USA; opechanova@mscl.msstate.edu; 2 Institute for Genomics, Biocomputing and Biotechnology, Mississippi State University, Mississippi State, MS 39762, USA

**Keywords:** proteomics, maize, pathogen, interaction, defense, resistance, mycotoxin, pathogenesis-related, *Aspergillus flavus*, *Fusarium* spp., *Curvularia lunata*, plant virus

## Abstract

Maize (*Zea mays* L.) is a host to numerous pathogenic species that impose serious diseases to its ear and foliage, negatively affecting the yield and the quality of the maize crop. A considerable amount of research has been carried out to elucidate mechanisms of maize-pathogen interactions with a major goal to identify defense-associated proteins. In this review, we summarize interactions of maize with its agriculturally important pathogens that were assessed at the proteome level. Employing differential analyses, such as the comparison of pathogen-resistant and susceptible maize varieties, as well as changes in maize proteomes after pathogen challenge, numerous proteins were identified as possible candidates in maize resistance. We describe findings of various research groups that used mainly mass spectrometry-based, high through-put proteomic tools to investigate maize interactions with fungal pathogens *Aspergillus flavus*, *Fusarium* spp., and *Curvularia lunata*, and viral agents Rice Black-streaked Dwarf Virus and Sugarcane Mosaic Virus.

## 1. Introduction

Maize is the most grown cereal crop in the world (990.64 million tons per year according to USDA WASDE report, May 2015) valued for its nutritional properties as food and feed, and as a source material for many industrial uses. Diseases inflicted on this important plant species by naturally occurring pathogens are detrimental to the economy and threaten the food supplies around the globe, especially in the regions where maize is a staple food commodity. In addition, fungal infection and consequent contamination with mycotoxins pose serious health hazards to both humans and animals, which can be directly infected by fungus or can be affected by consumption of contaminated food and feed. Among the mycotoxins, the aflatoxin B1 (product of *Aspergillus flavus*) is the most carcinogenic compound found in nature [1,2], and it represents the most dangerous agent the maize producers and consumers might potentially face.

For decades, researches around the world have been investigating the processes underlying the mechanism of maize pathogen recognition and the launch of effective defenses. Engaging the most advanced system biology approaches, including “omics” disciplines, such as genomics, transcriptomics, and proteomics, they have moved towards a better understanding of the many processes occurring during maize interactions with its pathogens. A thorough knowledge of molecular mechanisms of maize host response to pathogens is critical for the elucidation of a genetic basis of host resistance.

The advent of mass spectrometry (MS) application in proteomic research caused a true paradigm shift, allowing for many scientists to focus on the maize proteome. Continuous advancements of high-throughput technologies, availability of protein, EST and genomic databases (B73 genotype [3]) further accelerated the maize proteomics field.

In this review, we present a summary of proteome-based research of maize interaction with, and defense against, diverse pathogens. Various proteomic techniques were used, including both gel-based and gel-free approaches. Numerous potential protein and gene candidates in maize disease resistance were identified. Of special interest, these proteins could serve as selective markers for the development of resistant elite varieties, either through conventional breeding or via genetic engineering.

## 2. Maize-Pathogen Interactions from a Proteomics View

### 2.1. Maize vs. Aspergillus Flavus and Aflatoxins

*Aspergillus flavus* is a globally found fungal pathogen that causes disease in many agricultural crops and contaminates them with aflatoxins, very toxic metabolites that are produced via secondary metabolism [4,5]. Aspergillus ear rot caused by *A. flavus* is the major maize disease worldwide, and grains contaminated with aflatoxins present immense agronomical problems leading to more than one billion of dollars lost annually, according to The American Phytopathological Society [6]. Disease development can occur pre- and post-harvest and is most serious in the Southern United States, where hot weather and frequent droughts trigger aflatoxin production. If not controlled, aflatoxins might be present in a wide range of maize-based foods and feeds, as well as in dairy products. They pose serious health hazards to both humans and animals, if digested via contaminated food and feed. In humans, aflatoxins have been directly linked to hepatocellular carcinoma, since they are metabolized in the liver [7].

#### 2.1.1. Kernel Resistance

A notable number of proteomic investigations has been performed on maize kernel tissues [8,9,10,11,12,13,14,15,16,17]. Chen and colleagues examined numerous maize genotypes with dissimilar levels of resistance to aflatoxins accumulation. In one of their earliest studies using one-dimensional electrophoresis (1DE) and N-terminal sequencing, they identified a 14-kDa trypsin inhibitor that was significantly more abundant in kernel protein extracts from seven aflatoxin-resistant inbred lines, while not detectable or less abundant in kernels of six susceptible genotypes [13]. High constitutive levels of this protein are believed to be contributing to the maize kernels’ resistance as demonstrated by antifungal assays. Trypsin inhibitor purified from corn kernels caused spore rupture and abnormal hyphal growth of *A. flavus*. It also inhibited *A. flavus* growth via inhibition of fungal α-amylase, under certain experimental conditions [15]. Including other kernel tissues (endosperm and embryo), and applying more advanced proteomic tools such as two-dimensional electrophoresis (2DE) and tandem mass spectrometry (MS/MS), additional constitutive resistance-related proteins were identified [10,11,12]. Both embryo and endosperm of resistant lines (MP420, Mp313E, GT-MAS:gk, CI2, MI82, and T115) exhibited similar defense patterns and unique and/or higher expression (at least 5-fold) of several interesting proteins. These proteins can be grouped into the following categories: (i) storage proteins, which include globulin 1 and 2, and late embryogenesis abundant proteins (LEA3, LEA 14); (ii) stress-related proteins, such as aldose reductase (ALD), osmotic stress-related proteins WSI18, peroxredoxin antioxidant (PER1), cold regulated protein, anionic peroxidase, glyoxalase I protein (GLX I) and several small heat shock proteins (HSP); and (iii) antifungal proteins, such as trypsin inhibitor and pathogenesis-related (PR) protein 10 (PR-10). Antifungal PR-10 protein undoubtedly plays a role in host kernel resistance. Overexpression of *ZmPR-10* gene was found to be inhibitory to both *A. flavus* hyphal growth and conidial germination [14]. Furthermore, using RNAi gene silencing approach, fungal colonization and aflatoxin accumulation were more extensive in *pr10*-silenced transgenic kernels, while *pr10*-silenced callus lines displayed enhanced sensitivity to heat stress treatment, and significantly reduced *pr10* transcription.

Based on their observations, the authors proposed the existence of an association between stress tolerance and disease resistance [8]. LEA proteins, for example, are known to protect higher plants from dehydration caused by environmental stresses, especially drought [18]. In maize kernels, LEA proteins probably assist in aflatoxin resistance as well. Direct and active involvement of GLX1 in aflatoxin resistance was validated through the control of its substrate, methylglyoxal (MG), which induced aflatoxin production in infected kernels [11]. Few susceptible genotypes did exhibit significant increases in MG content when infected. The evidence of association between stress tolerance and disease resistance can be exploited in the future as a novel strategy to enhance maize resistance to fungal diseases.

#### 2.1.2. Rachis Resistance

Contamination of grains is of the highest concern for maize marketing and consumption. Therefore, a majority of the research is being devoted to this tissue. However, infection of other parts of the maize plant is equally important, as these organs may contribute to the resistance or susceptibility by affecting the pathogen’s behavior within a maize ear and/or the whole plant. For example, maize rachis (cob) plays a role in the delivery of nutrients to developing kernels, and it was reported that *A. flavus* uses it as a conduit for its spread within a maize ear [19,20]. When developing ears of aflatoxin-susceptible and aflatoxin-resistant hybrids were inoculated with GUS-tagged *A. flavus* strain, fungus moved freely throughout the maize cob and to the kernels in susceptible hybrids. In resistant hybrids, however, fungal spread was halted in rachis preventing it from entering the kernels [19]. The same phenomenon was observed in maize inbreds [20]. To elucidate the possible role of rachis in aflatoxin resistance, a comprehensive large-scale study was carried out by Pechanova *et al.* [21,22]. The two-dimensional Difference In Gel Electrophoresis (2D-DIGE) comparison of rachis from 21-day-old maize of four genetically unrelated maize inbreds (aflatoxin-resistant Mp313E and Mp420, and aflatoxin-susceptible SC212m and B73) revealed that resistant rachis contained 44 proteins of significantly higher abundance, mainly abiotic stress-related proteins such as heat shock proteins, antioxidant enzymes (superoxide dismutase (SOD), ascorbate peroxidase (APX), thioredoxin, *etc.*), and other diverse stress-related proteins. Proteins from the phenylpropanoid pathway, such as phenylalanine ammonia lyase (PAL), caffeoyl-CoA-3-*O*-methyltransferase1 and chalcone flavonone isomerase, were also present at considerably higher levels. Susceptible rachis, on the other hand, had significantly higher expression of 26 proteins, the majority of which were biotic stress-associated PR proteins, such as chitinases, basic endochitinase C and germin-like proteins (GLP) subfamily 1 member 17.

In regards to a fungal challenge, tremendous differences between resistant and susceptible genotypes were observed in response to *A. flavus*, especially after a long-term exposure. Thirty-five days post-infection, genotypes Mp313E and SC212m reacted with differential expression of 30 and 48 proteins, respectively. However, unlike resistant Mp313E, susceptible inbred SC212m exhibited a much more robust response via strong upregulation (3–10-fold) of fifteen different PR proteins including chitinases, glucanases, protein P21, permatin, PR-1, PR-5, PRm3, and PRm6b. The resistant genotype did not induce strong defenses as these proteins were already constitutively accumulated in its rachis, as shown in proteomic maturation studies (young *vs.* mature rachis). The most remarkable difference was observed for PRm3 (class III chitinase) that accumulated 40-fold during maturation of resistant rachis, as opposed to a 7.3-fold increase in the susceptible line. These findings indicated that rachis from resistant and susceptible genotypes differ in their defense mechanisms. While susceptible rachis relies on inducible defenses via action of antifungal proteins, resistant rachis is almost exclusively dependent on constitutive defenses. Abiotic stress-related proteins appear to be vital for young rachis to control oxidative stress caused by heat and drought, whereas biotic stress-responsive proteins, such as PR proteins, become more important as the ear matures. The subset of defense-related proteins including several isoforms of chitinases, PRm 6b, β-1.3-glucanase, GLP subfamily 1 member 17, catalase 3, Asr protein, abscisic stress ripening protein 1, auxin-binding protein 1, abscisic acid (ABA)-responsive protein, caffeoyl-CoA 3-*O*-methyltransferase 1, and remorin might be especially important for rachis resistance, as these proteins mapped near quantitative trait locus (QTL) for resistance to aflatoxin accumulation present on several chromosomes [22].

Similarly to kernels [13], association between abiotic stress tolerance and aflatoxin-resistance seem to be a crucial factor in rachis defense. This was the most obvious for two small HSPs that were almost 11-fold more abundant in resistant *vs.* susceptible rachis [22]. Unlike in susceptible inbreds, resistant rachis is able to cope better with heat and drought challenges via high expression of abiotic stress-related proteins, which simultaneously provide a good protection against *A. flavus*/aflatoxins [22].

#### 2.1.3. Silk Resistance

Comparative protein profiles of silk from maize genotypes (resistant Mp313E, Mp420, and susceptible SC212m, Mp339) were also investigated via 2DE [23]. Silk seems to play an essential role in pre-harvest contamination of maize, as it is easily accessible to the pathogenic agents, thus serving as a main point of entry and conduit for microbial infection. Once external silk of field-grown maize is colonized with *A. flavus*, fungal growth down the silk into the cob interior is rapid, and followed by subsequent colonization of kernel surfaces [24]. At the same time, silk very likely serves as a first line of defense against microbial infection, and its antifungal properties have previously been reported [25]. Three PR proteins could play a major role in *A. flavus*/aflatoxin resistance. Hydrolytic enzymes chitinase A, PRm3 chitinase, and chitinase I were constitutively expressed at higher levels in silks of both resistant maize varieties when compared to their susceptible counterparts [23]. These findings were supported by antifungal assay showing that extracts from resistant inbreds had higher antifungal activity than those from susceptible lines.

#### 2.1.4. Summary of Maize Protein-Based Defenses against *A. flavus*

Maize resistance to *A. flavus* infection and aflatoxin accumulation is a polygenic, quantitatively inherited trait strongly influenced by the environment [26,27,28].

**Figure 1:**
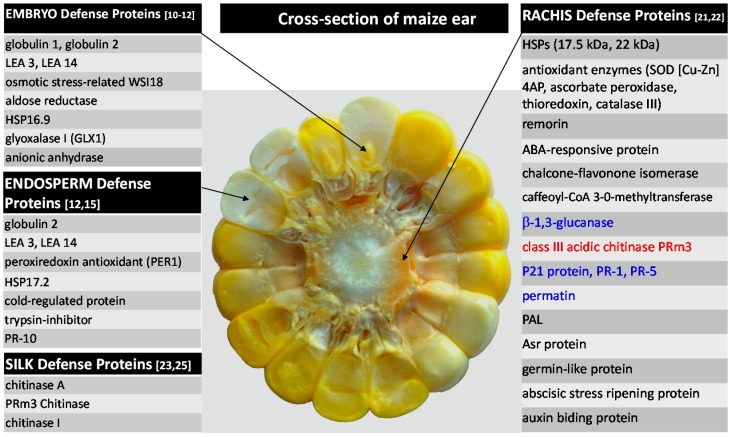
Selected proteins associated with maize defense against *Aspergillus flavus.* References are given in parenthesis next to the tissue-specific protein groups. Proteins in black and blue color represent constitutive and induced proteins, respectively. Protein in red was constitutively more abundant in resistant line, and induced in both resistant and susceptible lines (Modified from [29]).

As indicated from proteomic studies reviewed above, maize ear defense is correspondingly a very complicated mechanism consisting of diverse proteins, metabolites, and signaling molecules. It involves antifungal activity, host cells detoxification, cell wall reinforcement, primary and secondary metabolism, signaling, *etc.* Constitutive proteins seem to serve as major resistance factors, but inducible defenses are also essential. Figure 1 summarizes protein-based defenses of maize plant in tissues that are most instrumental to both infection and resistance by/against the *A. flavus*.

### 2.2. Maize vs. Fusarium Pathogens

*Fusarium* species represent another major group of fungal pathogens associated with maize diseases. This ubiquitous soil-borne genus contains several toxigenic species, with *F. graminearum* and *F. verticillioides* being the most common pathogens of maize. They can infect different parts of the maize plant in all stages of its development, causing significant reductions in maize yield and quality. Most importantly, under certain favorable environmental conditions during both pre- and post-harvest processing, these fungi produce harmful mycotoxins further diminishing maize crops [30,31]. The most common mycotoxins produced by toxigenic Fusaria include fumonisin, deoxynivalenol, and zearalenone. They contaminate maize grains, and therefore represent similar health hazard like aflatoxins.

#### 2.2.1. *F. graminearum* and Gibberella Ear Rot

Gibberella ear rot is caused by *F. graminearum* and results in moldy, mycotoxin-contaminated maize kernels. Infection is more prevalent in cool and wet weather, at the beginning stages of silking, and usually originates at the tip of an ear, followed by spreading towards the base [32]. Deoxynivalenol and zearalenone produced by this fungus are harmful to both humans and farm animals.

To evaluate defense processes occurring early upon encountering this pathogen, protein profiles of developing maize kernels of two inbred lines were examined 48 h post-inoculation [33]. In this large-scale study conducted over three growing seasons, 878 proteins were identified and quantified via iTRAQ MS/MS as differentially expressed between the mock- and fungal-treated kernels, and/or between the two inbred lines. Both genotypes, resistant (CO441) and susceptible (B73), exhibited changes in proteomes during both mock inoculation and pathogen challenge. Ninety-six proteins were differentially abundant in at least one of the treatments. They consisted of several major groups of defense proteins including PR proteins PR-10, chitinases, xylanase inhibitors (XIP), thaumatin-like protein, zeamatin precursor, GLPs, and peroxidase. Not only were these proteins among the most strongly induced ones, but also they were often at significantly higher levels in the resistant *vs.* susceptible genotype. Proteins PR-10, PRm3 chitinase, and PR-5 thaumatin, were also identified via 2DE approach in *Fusarium*-treated silks of B73 inbred, 48 h post-infection, playing role in silk defense [33]. Differential expression was also obvious for several important enzymes from secondary metabolism, especially the phenylpropanoid pathway. PAL, the protein catalyzing the first and committed step of the pathway, was more significantly induced in susceptible inbred (B73) when compared to CO441. Cinnamyl alcohol dehydrogenase, 4-coumarate-CoA ligase, and phenolic *O*-methyltransferase did not show significant induction in any of the inbreds, but they were more abundant in the resistant genotype CO441. From the terpenoid biosynthetic pathway, hydroxymethylbutenyl 4-diphosphate synthase (HDS) of the isoprenoid methylerythritol phosphate (MEP) pathway was found among the most *F. graminearum*-responsive proteins in both genotypes. HDS was also more abundant in resistant CO441. Finally, of the two chalcone flavonone isomerases that are involved in flavonoid biosynthesis, one was induced by fungal infection in both genotypes, while another was more abundant in CO441. Overall, resistant genotype CO441 exhibited higher levels of many defense-associated proteins than susceptible B73, under both mock- and fungus-treated conditions. However, B73 displayed stronger response to the *F. graminearum* than the resistant inbred.

#### 2.2.2. *F. verticillioides* and Fusarium Ear Rot

*F. verticillioides* causes Fusarium ear and stalk rot. Disease is prevalent, especially during hot and dry weather, both before and after harvest. Fumonisins produced by this species appear to be one of the most common maize-based food and feed contaminants, therefore they are of high importance to the producers [30,31]. Fumonisins are highly toxic and have been associated with several livestock and humans diseases including cancer [34,35].

Protection of seeds during germination is critical for plant propagation and ultimately, for survival of plant species. Defense responses during this vulnerable developmental phase were examined in 20-hour-old germinating maize embryos challenged with *F. verticillioides* [36]. Twenty-four hours after infection, proteins from fungus-infected *vs.* sterile embryos were compared via 2DE, using neutral and acidic extraction conditions. Several groups of proteins have shown changes in their expression. Detoxifying enzymes catalase 2, SOD, and glutathione-*S*-transferase (GST) were almost completely absent in extracts from sterile embryos, while present in fungus-infected embryos, likely in order to protect them from oxidative damage and xenobiotics. Proteins involved in protein synthesis, folding and stabilization such as small HSP 17.2, peptidylprolyl *cis*-trans isomerase, and cyclophilin were also more abundant in fungus-challenged embryos. Another up-regulated protein belonging to this group, eukaryotic translation initiation factor 5A (eIF-5A), might be in demand to facilitate synthesis of proteins needed for defense. Protein extracts from sterile and fungus-infected embryos were also compared using immunological reactions, which confirmed induction of P23 protein, and constitutive expression of β-1,3-glucanases and chitinases. Finally, carbohydrate metabolism in embryos also appeared to be altered by *F. verticillioides*. As seen from the expression of two glyceraldehyde-3-phosphate dehydrogenases (GAPDH) (cytosolic 1 and 2), and fructose-bisphosphate aldolase, fungus seems to repress glycolysis and activate gluconeogenesis.

#### 2.2.3. *F. verticillioides* and Extracellular Matrix

Besides its other biological functions, extracellular matrix (ECM) serves as a first line of defense during the plant-pathogen interaction. As such, ECM is critical for plant survival. Actions taken in this compartment trigger a complex series of events leading to the rapid defense responses at the infection site and throughout the plant. To gain an insight into this earliest host-pathogen encounter, a pathogen attack was simulated by treating cell cultures with elicitor molecules found in an attenuated fungal pathogen [37]. After a 6-hour-long treatment, they responded by activating three major events. The first event was characterized by elicitor-induced secretion of six putative XIP isoforms into the ECM culture medium. These defensive enzymes inhibit xylanases that fungi secrete during infection to hydrolyze the host’s cell walls [38]. The second event included rapid changes in the phosphorylation status of several apoplastic peroxidases, implying that the phosphorylation regulatory mechanism is very likely controlling this host-pathogen contact. More specifically, two peroxidases were consistently and significantly dephosphorylated within 10 min of *Fusarium* elicitor treatment, although their relative abundances remained unchanged. Finally, cell wall-enriched protein fractions contained considerably increased levels of cytosolic GAPDH and HSPs suggesting that during elicitor-induced ECM-mediated defenses these classical cytosolic proteins are specifically targeted to the cell walls. Furthermore, the three major elicitor-induced occurrences were accompanied by increased production of H_2_O_2_ as determined by histochemical staining. Accumulation of H_2_O_2_ is consistent with the onset of elicitor-induced oxidative burst, a central event in the establishment of an effective resistance response. Also observed was a disappearance of β-*N*-acetylglucosaminidase (β-NGase) protein spots. This protein hydrolyses bacterial cell wall peptidoglycan [39], and the authors speculate that the disappearance of β-NGase is likely the result of its oxidative cross-linking to the cell walls. Fungal elicitor-induced changes in maize ECM proteome thus strongly imply that this important anatomical structure plays a complex role in maize defense machinery.

#### 2.2.4. Summary of Maize Protein-Based Defenses against *Fusarium* spp.

**Figure 2:**
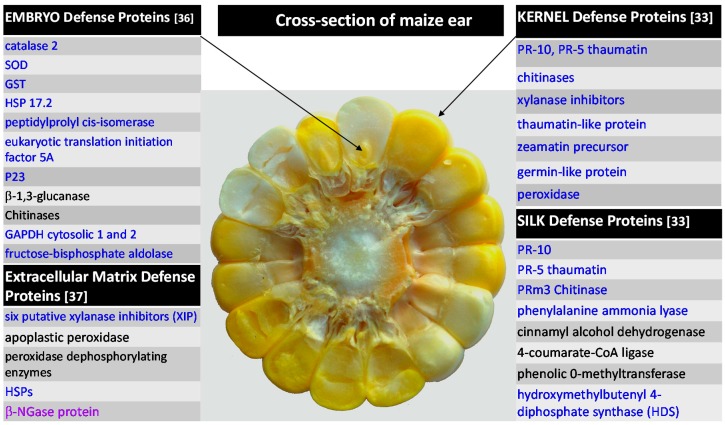
Selected proteins associated with maize defense against *Fusarium* spp. References are given in parenthesis next to the tissue-specific protein groups. Proteins in black and blue color represent constitutive and induced proteins, respectively. Protein in violet was not detected after the pathogen challenge.

Studies reviewed above strongly indicate that maize resistance to *Fusarium* spp. and *A. flavus* are comparable. Similar groups of proteins were found to be responsive against diseases caused by both types of fungi [22,33,36]. Suggestion of a common resistance mechanism between *Fusarium* ear rot/fumonisins and *Aspergillus* ear rot/aflatoxins was previously made from QTL mapping, which showed that some of the genes implicated in resistance to ear rots and mycotoxin accumulation were identical or genetically linked [40]. Furthermore, in very recent study [41], selected defense *pr* genesand proteins (catalase, superoxide dismutase, peroxidases) were monitored in developing kernels of resistant and susceptible maize genotypes inoculated with *F. proliferatum*, *F. subglutinans*, and *A. flavus*. Similarly to rachis tissue [22], higher gene transcription and enzymatic activities in uninoculated kernels of resistant line testify about constitutive defense against the pathogens. On the other hand, susceptible lines responded with induction of reactive oxygen species-scavenging proteins. In addition, maize 9-lipoxygenases (LOX) ZmLOX12 and ZmLOX3 suppressed contamination by *F. verticillioides* and *A. flavus*, respectively [42,43]. Hence, breeding for Aspergillus and Fusarium resistances may produce parallel outcomes. Figure 2 summarizes protein-based defenses of maize plant in tissues that are of interest in regards to infection by *Fusarium* spp.

### 2.3. Maize vs. Curvularia lunata

*Curvularia lunata* is a foliar fungal pathogen and a causal agent of Curvularia leaf spot of maize. It can cause damage to the maize yields, especially in hot and humid climates [44]. It has been reported that *C. lunata* produces a furanoid type toxin, both *in vitro* and *in planta*, which can possibly lead to leaf lesions [45]. Unlike Aspergilli and Fusaria, not much is known about *C. lunata* pathogenesis in maize at molecular level. Huang *et al.* attempted to unravel the pattern of resistance by comparing four maize inbreds ranging from highly resistant to susceptible to *C. lunata* [46,47]. Leaf proteomes analyzed 24 h post-infection revealed that resistant lines were much more responsive to infection than susceptible lines [47]. From 100 differentially expressed spots, only 8 were identified from 10 selected and analyzed spots. The expression levels ofGLP, translation initiation factor eIF-5A, oxygen-evolving complex (OEC), and two oxygen-evolving enhancer proteins (OEE 1, OEE 2) were found to be significantly more abundant in highly resistant variety Luyuan 92, when compared to the three other genotypes. GLPs, for instance, are ubiquitous developmentally regulated cell wall glycoproteins that possess a wide range of functions [48], including defense against pathogens [49,50]. Several GLPs have enzymatic activities of SOD and oxalate oxidase (OXO) producing hydrogen peroxide (H_2_O_2_), which can serve as a signaling molecule for a range of defense reactions, including cell death, and as a cofactor for cell wall strengthening by cross-linking [50,51]. In addition, GLPs often bind non-covalently to cell walls [52,53], potentially contributing to structural reinforcement. Consequently, GLPs might be able to modulate host resistance against pathogen by signaling via H_2_O_2_, and/or cell wall strengthening. This also may be the case for Luyuan 92 resistance to *C. lunata* but it remains to be confirmed in further studies. Similarly, Photosystem II proteins OEC and OEEs were previously reported as responsive to abiotic and biotic stresses [54,55], therefore they might act in the maize-*C. lunata* interaction as well. When longer viral exposures (24, 36, 48, 60 and 72 h) were examined, more proteins were identified as differentially expressed, especially in the resistant genotype 78599-1 [46]. The majority of these proteins were, nevertheless, common for both types of inbreds. Twenty-seven proteins that changed at least 3-fold in abundance were mainly related to photosynthesis, respiration, oxidative and drought stress, and signal transduction. Among them, putative glutathione peroxidase (GPX), APX and 22 kDa drought-inducible protein are the most common facilitators of plant tolerance to environmental challenges, as shown for maize-*A. flavus* interactions. By analogy, these proteins are also possible mediators between maize stress tolerance and resistance to *C. lunata.*

### 2.4. Maize vs.Viral Pathogens

Viral diseases are also fairly common in maize. They occur throughout the maize-growing regions around the world, and they can cause sporadic but very damaging epidemics. Proteomics of maize-virus interactions is a relatively new research area, and unlike for fungal pathogenesis, not much is known about the virus impact on the biology of a maize plant at the molecular, especially proteomic, level. Several recent studies [56,57,58] addressing the effect of viruses on maize protein expressions will be reviewed in the following paragraphs.

#### 2.4.1. Rice Black-Streaked Dwarf Virus

Li and colleagues scrutinized the long-term adjustment of the maize elite, but very susceptible line (Ye478), to Rice Black-streaked Dwarf Virus (RBSDV) [58]. This insect-transmitted pathogen is considered a major causal agent of maize rough dwarf disease (MRDD) in China, and it is responsible for dwarfed and severely damaged maize plants. Fifty days post-infection, virus-infected leaves responded with significant changes in abundance for 123 proteins, as revealed via 2DE and MS/MS. Functional variety of 91 identified proteins suggested an engagement of a large arsenal of biological processes and pathways, most likely due to severe morphological changes taking place upon viral encounter. For example, the accumulation of detoxifying enzymes peroxidase 39, APx2-cytosolic APX, and catalase isozyme 3 implies an onset of oxidative stress caused by RBSDV, and a subsequent need for regulation of cellular redox state. Higher levels of lipoxygenases and small GTP-binding proteins in virus-infected plants suggest that these proteins modulate the triggering of downstream defenses via signaling pathways.

UDP-glucosyltransferase BX9 catalyzes conversion of benzoxazinoids to their inactive glucoside form for their deposition to vacuoles. Benzoxazinoids are major grass phytotoxins that offer protection against a wide range of herbivores, fungi and bacteria [59]. Up-regulation of UDP-glucosyltransferase BX9 in RBSDV-infected plants indicates that these natural insecticides are very likely a part of maize defenses against RBSDV.

Consistent with severely altered leaf morphology, the upregulation of cinnamyl alcohol dehydrogenase and caffeic acid 3-*O*-methyltransferase may have led to modified cell walls in infected plants. These plants had rough leaves with enations on the veins. MRDD establishment is also followed by profound adaptation in carbohydrate metabolism, as numerous proteins from glycolysis, TCA cycle, glycogenesis, and pentose phosphate metabolism, were extensively altered in expression. Particularly evident was the decrease in transketolase levels in diseased plants, leading to a severely weakened influx of proteins affecting plant growth and development. On the other hand, elevated amounts of starch granules that were observed in MRDD-diseased leaves were very likely the result of enhanced expression of ADP-glucose phospohorylase small subunit in virus-infected plants. Finally, photosynthesis, carbon fixation and assimilation as well as starch synthesis also appeared intensified in virus-infected leaves [58].

Overall, the invasion by RBSDV causes tremendous changes in maize’s metabolism, which subsequently leads to severe morphological differences when compared to normal plants. During MRDD disease, numerous fundamental biochemical pathways become seriously compromised, which is accompanied by significant changes in expression of proteins, particularly those associated with plant growth. The proteomic study reviewed above clearly showed that the occurrence of MRDD results in very complex responses involving a number of proteins, as well as defensive and signaling molecules.

#### 2.4.2. Sugarcane Mosaic Virus

Sugarcane Mosaic Virus (SCMV) is another viral pathogen and causal agent of mosaic disease in maize and other graminaceous plants. SCMV occurs around the globe especially in regions where susceptible varieties are grown. Two proteomic gel-based investigations were conducted by Wu *et al.*, in which authors compared leaf protein profiles from resistant (Siyi) and susceptible (Mo17) inbred lines, 6 and 12 days post-infection (dpi) with SCMV, respectively [56,57]. With regard to functional classifications, similar groups of responsive proteins were observed in both genotypes for both time points. Ninety-six (6 dpi) and 93 (12 dpi) differentially expressed and identified proteins were predominantly from energy and metabolism, stress and defense, and photosynthesis, followed by protein synthesis and folding, signal transduction/transcription, and carbon fixation. However, a larger proportion of stress/defense-related and signal transduction/transcription-associated proteins were upregulated in the resistant genotype relative to the susceptible line, especially after longer viral exposure. In contrast, photosynthesis-associated proteins were down-regulated in the resistant inbred. After longer exposure to the virus, (12 dpi) enzymes from the major energy-producing glycolysis and gluconeogenesis pathways exhibited an increase in expression in the resistant line, while they were considerably lower in the susceptible maize genotype. Nineteen (6 dpi) and 17 (12 dpi) differentially expressed proteins were novel proteins that have not been previously known as virus-responsive. Few examples for 6 dpi treatment include remorin, glutamate dehydrogenase, calcium-dependent protein kinase, ATP synthase CF1 alpha subunit, and bZIP transcription factor ABI5. Abscisic stress ripening protein, serine/threonine-protein kinase, ferredoxin-NADP reductase, nucleolar RNA helicase 2, and 30S ribosomal protein S5 were among 17 newly identified proteins during 12 dpi experiment. More importantly, these proteins broaden the repertoire of possible virus-defensive maize proteins and may serve as good candidates for future resistance-related studies. Although similar biological processes in both lines were affected by infection, only 17 proteins were common for both inbreds. They include aconitase, transketolase, fructose-bisphosphate aldolase, nucleoside diphosphate kinase, thioredoxin, ABA stress ripening protein, GST, chaperonin, T-complex protein, Ribulose-1,5-bisphosphate carboxylase/oxygenase (RuBisCo) large subunit, RuBisCo subunit binding protein beta subunit, ferredoxin-NADP reductase, remorin, histidine triad nucleotide binding protein, cysteine synthase, β-d-glucosidase precursor and electron transporter protein. Similarly to RBSDV infection, there were dramatic changes in expression of proteins involved in carbohydrates metabolism in both genotypes, suggesting a significant shift of this metabolic pathway. Viral proteins were also found and identified in the susceptible line at 6 dpi confirming the presence of infection. In addition, resistance to SCMV seems to be also instituted via phytohormones-mediated defenses. Evidence exists that plant pathogens evolved a mechanism to modify the host’s hormone levels to establish pathogenicity [60,61,62]. Indeed, quantitative differences in concentrations of salicylic acid (SA), ABA, ethylene, jasmonic acid (JA) and azelaic acid (AZA) were observed between mock-inoculated and SCMV-inoculated plants, as well as between the resistant and susceptible plants.

Both studies brought valuable input into previously not much known maize–SCMV interactions. Identified proteins are subject for further research and possible resistance candidates. Summary of maize leaf proteins implicated in its defense against viral pathogens is given in Table 1.

**Table 1:** Examples of proteins associated with maize defense against viral pathogens.

Pathogen	Maize Genotype	Induced Proteins	Repressed Proteins	Reference
Black-Streaked Dwarf Virus	Ye478 Susceptible	Peroxidase 39, APx2-cytosolic ascorbate peroxidase, catalase 3, oxygenase, caffeic acid 3-*O*-methyltransferase, cinnamyl alcohol degydrogenase, UDP-glucose pyrophosphorylase, GAPDH, ADP-glucose phosphorylase, lipoxygenases, GTP-binding proteins, enzymes of starch synthesis, hotosynthesis, carbon fixation and assimilation	Enzymes of glycolysis, TCA cycle, glycogenesis, and pentose phosphate metabolism, particularly transketolase	[58]
Sugarcane Mosaic Virus	Siyi Resistant	Enzymes of glycolysis and gluconeogenesis, remorin, cysteine synthase, glutamate dehydrogenase, calcium-dependent protein kinase, bZIP transcription factor ABI5, serine/threonine-protein kinase, nucleolar RNA helicase 2, 30S ribosomal protein S5		[56,57]
Sugarcane Mosaic Virus	Mo17 Susceptible		Enzymes of glycolysis and gluconeogenesis, remorin, cysteine synthase, ATP synthase CF1 α subunit	[56,57]
Sugarcane Mosaic Virus	Siyi and Mo17	Stress/defense-related and signal transduction/transcription-associated proteins,aconitase, transketolase,nucleoside diphosphate kinase, abscisic stress ripening protein, chaperonin, T-complex protein, ferredoxin-NADP reductase, histidine triad nucleotide binding protein, β-d-glucosidase precursor, electron transporter protein	Photosynthesis-associated proteins, thioredoxin, glutathione *S*-transferase, RuBisCo large subunit, RuBisCo subunit binding protein α subunit	[56,57]

## 3. Commonly Responsive Proteins in Maize-Pathogen Interaction

Several groups of proteins were found commonly associated with the maize response to fungal and/or viral pathogens (Table 2). Their main functions will be discussed briefly in the following paragraphs.

### 3.1. Pathogenesis–Related Proteins

PR proteins represented the largest and most diverse group of proteins associated with maize resistance. Generally, PRs are inducible by a variety of pathogens via salicylic acid, jasmonic acid, or ethylene signaling, which leads into their accumulation at the site of the attack but also systemically at distant parts of the plant [63]. Many are expressed constitutively in healthy plants during growth and development. This large and diverse group of proteins classified into 17 families possesses a broad range of enzymatic activities rendering them indispensable for plant survival upon pathogen attack. An arsenal of PR proteins was found differentially expressed in maize genotypes associated with fungal diseases imposed by *F. verticillioides* [36], *F. graminearum* [33], and *A. flavus* [14,22,23], including chitinase and β-1,3-glucanases. These antifungal hydrolytic enzymes are perhaps the most important among PR proteins. They are ubiquitous in the plant kingdom, can be preformed or inducible, and often work synergistically. The mode of their action is degradation of chitin and glucans, major structural polysaccharides of fungal cell walls and exoskeletons of arthropods and nematodes [64,65,66,67].

**Table 2:** Commonly responsive protein groups during maize interactions with fungal and/or viral pathogens. Selected proteins are listed.

Protein Group	Elicited by Pathogen	Tissue	Proteins	References
Pathogen related proteins	*Af*, *Fv*, *Fg*	Embryo, silk, rachis, kernels	Chitinase, glucanase, trypsin and amylase inhibitor, peroxidase	[14,22,23,33,36]
Detoxifying enzymes	*Af*, *Fv*, *Fg*, *Cl*, RBSDV, SCMV	Leaves, foliage, embryo, silk, rachis, kernels	SOD, catalase, PER, thioredoxin, glutaredoxin, glutathione reductase, GST, dehydroascorbate reductase	[12,22,33,36,46,56,57,68]
Proteins involved in secondary metabolism	*Af*, *Fv*, *Fg*, RBSDV, SCMV	Rachis, leaves, ear	PAL, caffeoyl-CoA 3-*O*-methyltransferase, chalcone-flavonone isomerase, cinnamyl alcohol dehydrogenase, hydroxymethyl-butenyl 4-diphosphate synthase	[22,56,58]
Proteins involved in energy producing pathways	*Af, Fv,* RBSDV, SCMV	Rachis, leaves	GAPDH, ADP-glucose pyro-phosphorylase, UDP-glucose pyrophosphorylase, glucose phosphate isomerase, fructose-bisphosphate aldolase, transketolase	[22,33,36,37,47,56,57,58]
Proteins involved in protein synthesis, folding and stabilization	*Af*, *Fv*, *Fg*	Embryo, rachis, kernel	elF-5A/initiation of translation; HSPs, chaperonins, peptidylprolyl *cis*-trans isomerase, cyclophilin	[22,33,36,37,46,47,56]

*Af*—*Aspergillus flavus*; *Fv*—*Fusarium verticillioides*; *Fg*—*Fusarium graminearum*; *Cl*—*Curvularia lunata*; RBSVD—Rice Black-streaked Dwarf Virus; SCMV—*Sugarcane mosaicvirus*.

Other PR proteins important in maize fungal resistance included zeamatin and proteins P21 and P23. These are the members of PR-5 family that have a close resemblance to a sweet-tasting protein thaumatin and are also known as thaumatin-like proteins (TLs). They permeabilize fungal cell walls causing a rapid cell lysis and killing the pathogen [69]. Others have bifunctional trypsin/α-amylase inhibitor activities that impair fungal germination [70]. Highly basic PR-5 protein P23 was induced in maize embryo infected by *F. verticillioides* [36] and highly acidic protein P21 in rachis infected by *A. flavus* [22]. Finally, both silks and kernels infected with *F. graminearum* responded with up-regulation of zeamatin and thaumatin, respectively [33].

A class III peroxidase belonging to the PR-9 group was also found as fungus responsive. ZmPrx16 increased in abundance in response to *F. graminearum* in kernels of both resistant and susceptible inbred, and it was also constitutively expressed at higher levels in resistant kernels [33]. Class III peroxidases are well-known defense-associated enzymes that are located in plant cell walls and vacuoles [71,72]. They create a physical barrier in host tissues to halt pathogen invasion by catalyzing the H_2_O_2_-dependentcross-linking of cell wall components resulting in enhanced lignification or suberization.

### 3.2. Detoxifying Enzymes

One of the most critical events upon plant infection is pathogen recognition followed by rapid apoplastic production of reactive oxygen species (ROS) via a process known as the oxidative burst [73,74]. The most common reactive ROS include superoxide, hydroxyl radical and hydrogen peroxide, and their induction is imperative for the outcome of host-pathogen interactions as they mediate the launch of diverse defensive systems. ROS serve as substrates for strengthening of host cell walls via oxidative cross-linking of glycoproteins and as secondary messengers for activation of molecular and physiological responses in plant cells. Although their induction is transient, ROS are extremely reactive, and if not controlled they can cause detrimental oxidative damage to the host cells that can lead to their death. Detoxification of ROS is therefore paramount to the cell survival. For this purpose, plants are equipped with effective ROS-scavenging systems consisting of detoxifying enzymes that are responsible for the maintenance of the steady state levels of ROS in different cell compartments. Key detoxifying enzymes found in plant cells include APX [22,74,75,76], glutathione peroxidase (GPX) [46,74,77], SOD [22,36,74,76,77,78], catalase [25,36,41,58,68,77], peroxiredoxin (PER) [10,11,12,57,74,79], thioredoxin [22,56,57,74], glutaredoxin [74,80], and glutathione reductase [74,76,81], and dehydroascorbate reductase [58,82], and their inducibility or repression was a common characteristic feature following exposure of maize to multiple stressors such as *F. verticillioides* [36], *F. graminearum* [33], *A. flavus* [22,68], *C. lunata* [46]. RBSDV [58], and SMV [56]. Catalase 2 and SOD, for example, were two main enzymes protecting maize embryo from oxidative damage during Fusarium ear rot establishment [36], and combined action of GPX and APX seems to be required for supporting maize defense response against *C. lunata* in both resistant and susceptible varieties [46]. Catalase activity was also significantly higher in immature embryos of maize genotypes resistant to *A. flavus* infection, while the challenge by the pathogen caused further increased in catalase activity [68]. Antioxidant enzymes SOD [Cu–Zn] 4AP, peroxiredoxin, and thioredoxin-like protein 5 were also found constitutively expressed at higher levels in multiple tissues of aflatoxin-resistant maize varieties [12,22], likely contributing to the maize tolerance to the heat and drought which renders them better protection against aflatoxin production by *A. flavus*.

With regard to viral pathogens, APx2-cytosolic ascorbate peroxidase and catalase isozyme 3 were accumulated in RBSDV-infected leaves suggesting that these proteins protect cells from toxicity of ROS [58]. Finally, distinct antioxidant activities were observed in SCMV-infected leaves. SOD was down-regulated in susceptible maize while ascorbate peroxidase was up-regulated resistant line [56].

In addition to ROS, plants also detoxify other contaminants by conjugating them to diverse small non-protein antioxidants such as glutathione. These reactions are catalyzed by GSTs, a family of metabolic isozymes that are known to be highly expressed in major cereal crops representing up to 2% of their total foliage proteome [83]. They have been implicated in maize detoxification of herbicides [84,85], and during maize infection with *F*. *verticillioides* [36] and *A. flavus* [22]. The xenobiotic detoxifier GST14 was the most upregulated protein (6.9-fold) in maize rachis after a 35 days exposure to *A. flavus* [22].

### 3.3. Proteins Involved in Secondary Metabolism

Higher plants harbor a wide spectrum of organic compounds that perform a myriad of cellular roles including protection against predators and pathogenic microorganisms. They are produced via secondary metabolism, predominantly the phenylpropanoid, the isoprenoid and the alkaloid pathways [86]. The phenylpropanoid pathway in particular supplies plant cells with a variety of phenolics with a broad range of defensive properties. Some act as toxins, for example phytoalexins and isoflavonoids with antimicrobial and antifungal properties, respectively.

Maize 9-LOX converts linolenic and linoleic acid into 10-oxo-11-phytodienoic acid (10-OPDA), which acts as a phytoalexin, inhibiting growth of *A. flavus* and *F. verticillioides* [87]. Direct toxicity of phenolics to *A. flavus* is also probable. It was previously reported against *A. flavus* [88] and *Fusarium* spp. [89,90]. Other phenolics are precursors of lignins, major components of plant secondary cell walls where they are responsible for wall’s mechanical rigidity [91].

Although proteomic studies on maize-pathogen interaction did not aspire to identify stress-inducible secondary metabolites, proteins responsible for their production were of great interest as their induction indicates involvement of chemical defenses. For example, PAL, a key regulatory enzyme of the phenylapropanoid pathway, together with caffeoyl-CoA 3-*O*-methyltransferase1 and chalcone–flavonone isomerase were up to 5-fold higher in rachis from aflatoxin-resistant maize genotypes [22]. This observation implies that the impediment of *A. flavus* spread within an ear of resistant genotypes may be likely a consequence of increased cell wall lignification [19,20]. Cinnamyl alcohol dehydrogenase is another example of a cell wall reinforcing protein, catalyzing the final step in a branch of phenylpropanoid synthesis that leads to the production of lignin monomers. The enzyme was inducible upon viral infection where it was preferentially accumulated in the leaves of plants diseased with RBSDV [58] as well as with SCMV [56]. Thus, fortification of cell walls via enhanced lignin biosynthesis appears to be a common mechanism of resistance against fungal and viral disease.

### 3.4. Proteins Involved in Energy-Producing Carbohydrate Metabolic Pathways

Recognition of pathogen and subsequent deployment of downstream defenses is a complex process requiring immense reprogramming within plant cells that involves generation of signaling molecules, cell wall reinforcements, cytoskeleton rearrangement, induced synthesis of proteins, secondary metabolites, *etc.* Needlessly to say, all these processes have high demand for energy influx that is predominantly provided by primary metabolic pathways [92,93,94,95]. In maize-pathogen interactions, carbohydrate metabolism was one of the maize primary metabolic processes impacted the most by pathogen infection. Response-associated proteins included enzymes from glycolysis, gluconeogenesis, pentose phosphate pathway, TCA cycle, as well as associated mitochondrial electron transport and ATP biosynthesis [22,33,36,37,46,56,57,58]. Whether these pathways serve solely as the energy providers for costly maize defenses or they also participate in resistance, will need to be further inspected. There is evidence demonstrating that plants also use carbohydrate metabolism as a source of signaling molecules to directly or indirectly activate downstream defenses [94]. Plant GAPDHs, for example, have been known for some time to have non-traditional activities that are beyond the catalysis of energy production by glycolysis. In *Arabidopsis*, cytosolic GAPDH can be reversibly inactivated by H_2_O_2_ followed by reactivation with reduced glutathione, thus mediating signaling via ROS [96,97]. In maize, GADPH was upregulated in response to *A. flavus* [22] while it was lowered in expression after *F. verticillioides* infection [36]. The most robust impact on carbohydrate metabolism was seen in maize infected with viral agents. ADP-glucose pyrophosphorylase, UDP-glucose pyrophosphorylase, glucose phosphate isomerase, GAPDH, fructose-bisphosphate aldolase, and transketolase were all dramatically altered upon infection with RBSDV [58], the agent that causes immense changes in maize physiology. Similarly, major energy-producing pathways of carbohydrate metabolism were significantly compromised in maize infected by sugarcane mosaic virus, especially after long viral exposure of a susceptible line [56,57].

Given that simple sugars are actively involved in signaling [98] and gene regulation [99], their concentration in plant cells under a biotic stressor may directly or indirectly correlate with a plant’s capability to trigger adequate defenses. Nonetheless, functional studies are necessary to fully clarify how alterations in carbohydrate metabolism impact pathogen establishment, disease development and maize resistance.

### 3.5. Proteins Involved in Protein Synthesis, Folding and Stabilization

This group of proteins implicated in maize-pathogen interaction most commonly consisted of eukaryotic translation initiation factor 5A (eIF-5A), multiple HSPs, chaperonins, peptidylprolyl *cis*-trans isomerases, and cyclophilin [22,33,36,37,46,47,56]. Eukaryotic translation initiation factor 5A is best known for the initiation of eukaryotic cellular protein biosynthesis, but in recent years, it has also been found as pathogen-responsive. Its exact role in maize-pathogen interaction remains to be solved, but in other plant species, the function of eIF-5A is slowly becoming better defined. As demonstrated in *Arabidopsis*, eIF-5A can serve as a key element in regulating the induction of programmed cell death caused by infection with a virulent pathogen [100]. HSPs, on the other hand, are known stress proteins inducible under heat and other environmental stimuli [101]. As intra-molecular chaperones HSPs prevent the aggregation of newly synthesized proteins and the accumulation of proteins damaged by stresses [102]. Hence, they are responsible for maintaining proteins’ functional conformation essential for their cellular activities and ultimately for cell survival under stress [103]. These proteins seem valuable for maize resistance to *A. flavus* and aflatoxins, especially in southern states of the USA whose climate conditions facilitate aflatoxin production. HSPs were one of the most differentially expressed proteins with significantly higher levels (11-fold) in rachis tissues from aflatoxin-resistant maize genotypes [22] thus helping resistant lines to alleviate heat stress and subsequent control of aflatoxin production. Peptidylprolyl *cis*-trans isomerases (PPIs) and cyclophilins have similar roles as HSPs. They are upregulated under stress to accelerate folding or refolding of nascent or damaged proteins, respectively [104,105,106,107], showing that they are critical for cell adaptation under stress conditions.

## 4. Concluding Remarks

The presented review outlines the impact of several fungal and viral pathogens on the biology of maize, the world’s leading cereal crop. As seen from the compiled proteomic-based evidences, maize interaction with pathogens is a remarkably complex system engaging multiple levels of defense occurring in multiple tissues. This is anticipated since maize resistance to the majority of diseases is a polygenic quantitatively controlled trait that is strongly influenced by environmental conditions [108]. Therefore, the full comprehension of events underlying maize-pathogen interaction is a difficult task requiring a system biology approach. Future functional studies are still needed to fully unravel this intricate and complex defensive network that will help geneticists and breeders to develop resistant maize varieties.
